# Dynamic Loading—A New Marker for Abdominal Aneurysm Growth?

**DOI:** 10.3390/medicina59020404

**Published:** 2023-02-19

**Authors:** John Friesen, Lucas Stein, Farzin Adili, Peter F. Pelz

**Affiliations:** 1 Chair of Fluid Systems, Technical University Darmstadt, 64287 Darmstadt, Germany; 2 Division of Vascular and Endovascular Surgery, Klinikum Darmstadt, 64283 Darmstadt, Germany

**Keywords:** aneurysm, aneurysm modeling, pulse tracking

## Abstract

The growing possibilities of non-invasive heart rate and blood pressure measurement with mobile devices allow vital data to be continuously collected and used to assess patients’ health status. When it comes to the risk assessment of abdominal aortic aneurysms (AAA), the continuous tracking of blood pressure and heart rate could enable a more patient-specific approach. The use of a load function and an energy function, with continuous blood pressure, heart rate, and aneurysm stiffness as input parameters, can quantify dynamic load on AAA. We hypothesise that these load functions correlate with aneurysm growth and outline a possible study procedure in which the hypothesis could be tested for validity. Subsequently, uncertainty quantification of input quantities and derived quantities is performed.

## 1. Introduction

An aneurysm is a permanent focal dilatation of an artery that has increased in diameter by at least 50%. An abdominal aortic aneurysm (AAA) is present when the aortic diameter between the renal and common iliac arteries exceeds 30 mm. These lesions may be the result of several degenerative processes, including inflammatory, infectious, genetic, and traumatic conditions. AAAs lead to 1–2% of all deaths among men over 65 in western countries with an overall mortality of 80–90% after rupture [1].

Most AAAs are detected incidentally through diagnostic imaging techniques such as ultrasonography, whereupon the patient is checked at regular intervals [2]. To decide if a specific aneurysm should be treated or further observed, the clinician needs evaluation criteria. The diameter of the aneurysm and the expansion rate serve as the main indicators to make that decision. An intervention is typically planned if the maximum diameter exceeds 50 mm in women and 55 mm in men or if the AAA grows faster than 10 mm/year [3].

Nevertheless, there are ruptured aneurysms which do not fulfill the above-mentioned criteria. While 13% of AAAs with a diameter smaller than 50 mm rupture, 60% with a diameter greater than 50 mm stay intact [4]. This fact raises the question of how the rupture risk of a specific aneurysm should be evaluated and best be predicted.

A variety of biomechanical modelling approaches exist in the literature that aim to predict rupture risk more accurately than the diameter criterion. Alternative risk indicators are the peak wall stress (PWS), the peak wall rupture index (PWRI), and the probabilistic rupture index (PRRI), among others.

In general, these indicators determine wall stress distribution and their peak value (PWS) occurring in the AAA and compare them to wall strength (PWRI and PRRI) [5,6,7]. Several studies report an advantage of PWS, PRWI, and PRRI over maximum diameter while some studies show opposing results regarding PWS [8,9,10]. Biomechanical models for risk assessment require computed tomography angiography (CTA) or magnetic resonance angiography (MRA) as imaging modalities and subsequent finite element analysis (FEA) of the vessel wall, which has made their integration into clinical practice difficult to date [11]. Another option discussed for incorporating computer simulations into aneurysm evaluation are blood flow simulations. These simulations provide information about the stress generated by the friction of the blood against the vessel wall. However, the results have large uncertainties due to the strong assumptions made in running the simulation. However, to adequately represent the flow and thus calculate the forces on the vessel wall, it is necessary to have high-resolution information (both spatial and temporal) about the flow field. The acquisition of these boundary conditions for the flow simulation is currently not yet possible [11]. A comprehensive discussion of the advantages and disadvantages of different methods to evaluate aneurysms is presented by Friesen et al. [11].

In addition to biomechanical modeling approaches, biological surrogate markers are also of interest in indicating AAA progression. A variety of biomarkers exist that are associated with matrix degrading, inflammatory, and thrombotic processes in the AAA, including levels of matrix metalloproteases (MMP-2 and MMP-9), plasmin–antiplasim complexes (PAP), and D-dimers [12,13]. While circulating markers are relatively easy to determine, it is difficult to specifically link them to AAA, as other pathologies may also lead to increased levels of the corresponding markers.

This indicates that there is still potential for the development of new biomechanical markers, which would allow patient-specific rupture risk assessment while being applicable in a clinical setting. At the same time, biological markers should also be considered when exploring new comprehensive risk assessment methods.

We aim to incorporate two features in our modeling approach: the use of extensive patient-specific data via continuous measurement of blood pressure and heart rate and the description of AAA progression based on varying load and energy, both pressure- and frequency-dependent. To investigate the association of these variables with AAA progression, we aim to correlate them with established markers, such as AAA growth, and with promising biological markers, such as D-dimer concentration.

## 2. Hypothesis

Aneurysm growth follows complex processes that have not yet been fully understood, in which the wall structure interacts with hemodynamics and is subject to growth and remodelling processes (G&R) [3]. Still, the resulting problem is a locally varying degradation of the wall structure as a result of AAA progression until the vessel wall can no longer accommodate the forces acting on it and eventually ruptures.

Our approach is inspired by a model from strength theory. The strength of a material under cyclic stress is determined by means of the Wöhler test. In this test, the specimen is subjected to cyclic loading under a defined load. Both the mean stress and the nominal stress influence the number of tolerable load cycles. To determine a Wöhler, or S-N curve, either the mean stress or the stress amplitude is varied between each test cycle.

If a structure is dynamically loaded, then the damage model should appropriately represent this type of loading. As the mean stress and the stress amplitude increase, the load on the structure also increases.

Blood pressure is known to exert an influence on aneurysm development, and recent models have attempted to incorporate blood pressure values into risk assessments [14]. To our knowledge, however, the dynamic nature of blood pressure has not been considered to date.

Therefore, we hypothesize that fluid-mechanical loads acting on the arterial wall could be a predictor of aneurysm progression and correlate positively with AAA expansion rate. In accordance with this hypothesis, the objective is to define a suitable measure of the characteristic load on the aneurysm wall. We follow two approaches: the product of blood pressure and heart rate as a measure of dynamic load on the aneurysm wall, and an estimate of the volume-specific elastic deformation energy of the aneurysm wall as a predictor for aneurysm growth.

### 2.1. Dynamic Load in the Arterial System

The following assumptions are first made about the stresses in the arterial vascular system:

- In arterial blood vessels, mean arterial blood pressure (MAP) results in a mean stress on the vessel wall.
- The pressure pulse dynamically stresses the vessel wall; thus, the difference between systolic and diastolic blood pressures results in alternating stresses on the vessel wall.
- The heart rate contains information about the number of load cycles in a defined time interval and about the dynamics of the load, i.e., the slope of the pressure curve.

Given these assumptions, we now characterize the dynamic load acting on the arterial wall as a function of mean arterial pressure, pressure pulse, and heart rate. Writing the dynamic load as a continuous function of blood pressure over time would be more accurate, but as we aim to use measures that are established in clinical practice and can be recorded over a long time; therefore, discrete variables of blood pressure and heart rate seem better suited.

Remembering that the MAP can be estimated from systolic and diastolic pressure, a general approach to define a load increment $K_{i}$ acting on the arterial wall at a certain point in time is a linear combination of diastolic blood pressure, systolic blood pressure, and the product of pulse pressure, heart rate, and measuring interval:

(1) $$\begin{array}{cc} K_{i} & =MAP_{i}+\Delta p_{i}f_{i}\Delta t_{i}\\ \; & =\frac{1}{3}p_{s,i}+\frac{2}{3}p_{d,i}+\left(p_{s,i}-p_{d,i}\right)f_{i}\Delta t_{i}\\ \; & =p_{s,i}\left(\frac{1}{3}+f_{i}\Delta t_{i}\right)+p_{d,i}\left(\frac{2}{3}-f_{i}\Delta t_{i}\right). \end{array}$$

The total load acting on the arterial wall is then determined as a function of the incremental loads, in a straightforwad way, as their sum:

(2) $$K=\sum_{i=1}^{n}K_{i}$$

### 2.2. Elastic Deformation Energy

The second approach makes use of the volume-specific elastic deformation energy of the vessel wall as a predictor of aneurysm rupture risk. During each heartbeat, the vessel wall expands in systole, storing blood. The restoring forces cause the vessel wall to contract again in diastole and distribute the stored blood in the arterial system. Elastic energy is entered into the vessel wall with each heartbeat. Knowing the blood pressure, the heart rate, and the deformation of the vessel between two points in time, the energy input into the structure can be estimated. The energy input thus contains information about the load on the vessel wall in the form of blood pressure and heart rate, as well as information about the response to the acting load through the elastic deformation of the vessel wall.

We make a simplified estimate of the volume-specific elastic deformation energy in a given time interval via the pulse pressure and the deformation of the AAA between diastole and systole. Assuming linear elastic, homogeneous, and isotropic behaviour, we can substitute deformation with the quotient of pressure and pressure–strain modulus, a measure for local arterial stiffness. This is useful as we can measure the blood pressure continuously over a long period of time, whereas the elastic deformation of the AAA can only be determined by an ultrasound measurement at every medical examination. The incremental volume-specific energy is then determined by the blood pressure and heart rate at measuring time *i* and the stiffness parameter $E_{p}$ at examination time *j*.

(3) $$\begin{array}{cc} w_{i} & =\frac{1}{2}\Delta p_{i}\epsilon_{i}f_{i}\Delta t_{i}=\frac{1}{2}\frac{\Delta p_{i}^{2}}{E_{p,j}}f_{i}\Delta t_{i}\\ \; & =\frac{1}{2}\frac{{\left(p_{s,i}-p_{d,i}\right)}^{2}}{E_{p,j}}f_{i}\Delta t_{i} \end{array}$$

(4) $$E_{p,j}=\frac{\left(p_{s,j}-p_{d,j}\right)D_{d,j}}{D_{s,j}-D_{d,j}}$$

The stiffness parameter $E_{p,j}$ is determined from the measured AAA diameter at diastole $D_{d,j}$ and systole $D_{d,s}$, and the corresponding blood pressure $p_{d,j}$ and $p_{s,j}$, measured at the same time. The total specific energy input into the aneurysm is again determined from the sum of incremental energy inputs:

(5) $$w=\sum_{i=1}^{n}w_{i}$$

## 3. Testing the Hypothesis

**Figure 1:**
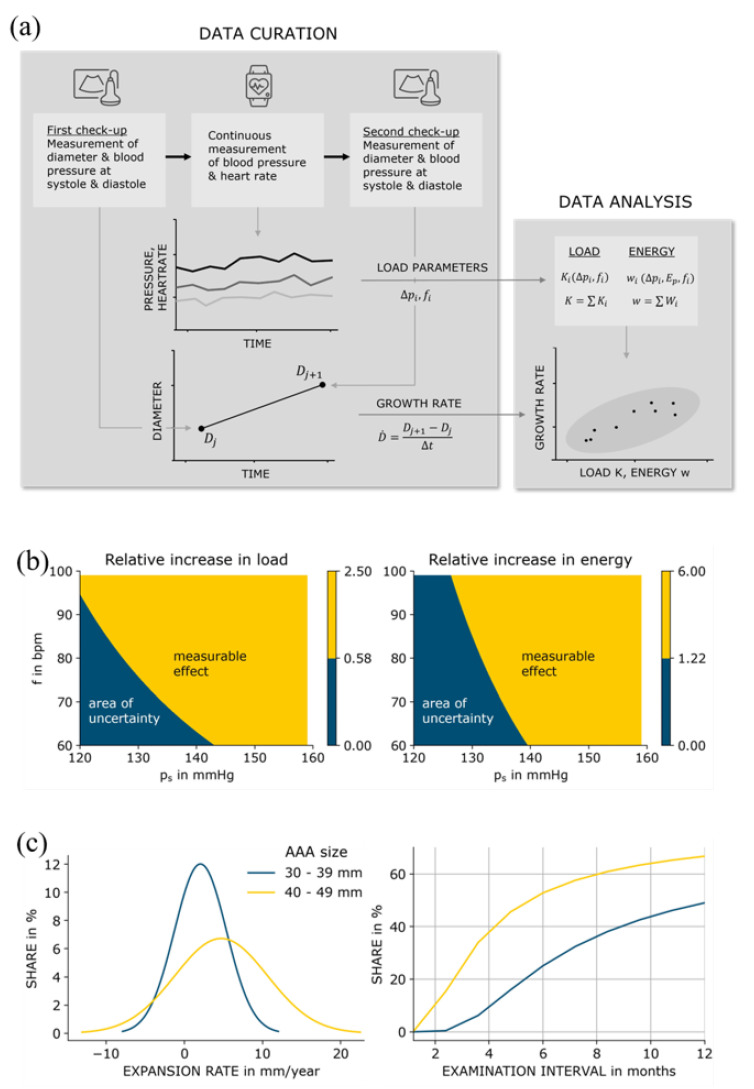
(**a**) Study framework; (**b**) relative load increase (**left**) and energy increase (**right**) depending on blood pressure and heart rate; (**c**) probability density function of AAA expansion rates corresponding to mean values and standard deviations of group 1 and group 2 given in [15] (**left**), and measured expansion rates that fall outside the measurement uncertainty of 2.16 mm in relation to examination interval (**right**).

The framework we propose is shown in Figure 1. To test our hypothesis, it is necessary to quantify AAA progression. We take the AAA expansion rate to be a predictor for aneurysmal progression and rupture risk and examine the extent to which it correlates with the load and the energy entered into the vessel wall.

For this purpose, patients with AAA will be followed in a prospective study. The study participants undergo regular examinations in which the diameter of the AAA and the elastic deformation between diastole and systole are measured. The expansion rate is determined as the change in diameter between two consecutive examinations divided by the respective time interval. The aneurysmal stiffness is determined with the pressure–strain modulus from the elastic deformation of the AAA and the pulse pressure at the time of the measurement.

Between the examinations, the participant’s blood pressure and heart rate are continuously recorded with a mobile measuring device. The incremental load and energy inputs are calculated from the measured blood pressure and heart rate, averaged over their respective time interval, and the total load and energy input are determined from the sum of incremental inputs between two examinations.

## 4. Uncertainty Quantification

The quality of the biomechanical markers *load* and *energy*, respectively, can only be as good as the quality of the data used to calculate their values. Both the load input and the volume-specific deformation energy are derived variables and functions of input variables that are subject to uncertainty. The uncertainty of the input variables is propagated with the derived variables.

First, for the input variables, relevant measurement methods and their uncertainty are considered. Then, the propagation of the uncertainty to the derived variables load, volume-specific deformation energy, and AAA expansion rate is examined. Thus we determine the influence that the uncertainty associated with the respective measurement method has on the result, in order to estimate which order of magnitude the correlation to be investigated should have at least.

### 4.1. Capturing the Measurands

#### 4.1.1. Blood Pressure

Non-invasive blood pressure measurements are usually performed using a blood pressure cuff, automatically by oscillometric measurement or manually by a physician using the Riva-Rocci method. Twenty-four-hour blood pressure measurements are usually performed with a portable cuff blood pressure monitor. For continuous blood pressure measurement over a period of several months, cuff devices do not offer sufficient comfort and are therefore not an option.

A promising alternative is blood pressure monitors that use optical measurement at the wrist by photoplethysmography (PPG) to analyze the change in blood volume and determine the blood pressure from it. This measurement method uses the same raw data that are used for optical heart rate measurement by PPG, but requires complex signal processing and analysis. The measuring devices must be calibrated at regular intervals by means of a blood pressure measurement using a cuff device [16].

Our reference device is the so-called *aktiia* wristband (Aktiia SA, Switzerland), which allows the continuous measurement of blood pressure using PPG, i.e., up to 14 measurements per day, and is approved as a medical device on the European market. The accuracy is 0.46±7.75 mmHg for systolic blood pressure measurement and 0.39±6.68 mmHg for diastolic blood pressure measurement according to the approval study. The permitted limits for non-invasive blood pressure measuring devices are 5±8 mmHg for systolic and diastolic blood pressure [17]. The *aktiia bracelet* measures blood pressure only when the wearer is at rest, as accuracy decreases during spikes in blood pressure and heart rate.

#### 4.1.2. Heart Rate

The gold standard for heart rate measurements is the electrocardiogram (ECG). Mobile heart rate monitors record the frequency via skin electrodes in a chest belt or via optical measurement using PPG on the wrist. There are a large number of available wearables on the market, such as fitness trackers and smartwatches, for heart rate measurement. Nelson’s 2019 comparative study, which examines the accuracy of a 24-h heart rate measurement by the Apple Watch 3 and Fitbit Charge 2, is used as reference. The accuracy is determined in comparison to the reference method, an ambulatory ECG system. The accuracy of the Fitbit Charge 2 is reported as −3.47±6.17 bpm [18].

#### 4.1.3. AAA Diameter

For the recurring measurement of the aneurysm diameter, only ultrasound is considered due to the radiation exposure of the more accurate computed tomography. A special feature of ultrasound measurements is the operator dependency, which leads to a systematic uncertainty in the measurement. Therefore, the measurement conditions and the method should always be specified and carried out by the same operator. Studies from 1991 to 2011 on intra- and interoperator variability of ultrasound measurements are considered [19]. Intraoperator variability refers to the measurement deviation in successive measurements by the same operator, interoperator variability refers to the measurement deviation in measurements by different operators. In general, intraoperator variability is lower, i.e., measurements by the same operator deviate less from each other and do not scatter as much as measurements by different operators. The study with the most accurate results gives the standard deviation for successive measurements by the same operator as 0.78 mm [20].

#### 4.1.4. Stiffness

A local measure of stiffness can be determined by the deformation of the AAA wall between diastole and systole and the pulse pressure acting upon the wall at that time [21]. An established method measures the elastic deformation of the arterial vessel by means of ultrasound and an echocardiographic system and, at the same time, the blood pressure by cuff measurement on the arm.

In the study from Länne, two stiffness values are determined for measurement at rest and during isometric strength exercise, since the stiffness of the vessel wall increases sharply as blood pressure rises. The pressure–strain curve is non-linear and has a inflection point between 90 and 110 mmHg. The course of both parts of the curve is approximately linear. The accuracy of the pressure–strain modulus when measured at rest is given as $0.75\pm 0.26\times 10^{5}\;{N/m}^{2}$ [22].

### 4.2. Uncertainty Propagation

We follow the methodology of the Guide to the Expression of Uncertainty in Measurement (GUM) [23]. In doing so, the input variables were first identified and characterized by their respective mean value and their uncertainty in the previous section. The law of uncertainty propagation according to Gauss can be applied if the model behaves sufficiently linearly in the range of the given variances and the input measurands do not correlate with each other.

Accordingly, the uncertainties of the combined quantities can be determined as follows:

(6) $$u\left(Y\right)=\sqrt{\sum_{i=1}^{N}{\left(\frac{\partial f}{\partial X_{i}}\right)}^{2}u^{2}\left(X_{i}\right)}$$

#### 4.2.1. Load Variable

For the load variable, this leads, according to Equations (1) and (6), to the combined uncertainty

(7) $$\begin{array}{cc} u(K) & =\{{\left(\frac{1}{3}+f\Delta t\right)}^{2}u^{2}\left(p_{s}\right)+{\left(\frac{2}{3}-f\Delta t\right)}^{2}u^{2}\left(p_{s}\right)\\ \; & +{\left(p_{s}\Delta t-p_{d}\Delta t\right)}^{2}u^{2}\left(f\right)+{\left(p_{s}f-p_{d}f\right)}^{2}u^{2}\left(\Delta t\right){\}}^{\;1/2}. \end{array}$$

#### 4.2.2. Energy Variable

For the energy variable, this leads, according to Equations (3) and (6), to the combined uncertainty

(8) $$\begin{array}{cc} u(W) & =\{{\left(\frac{p_{s}-p_{d}}{E_{p}}f\Delta t\right)}^{2}u^{2}\left(p_{s}\right)+{\left(\frac{p_{d}-p_{s}}{E_{p}}f\Delta t\right)}^{2}u^{2}\left(p_{d}\right)\\ \; & +{\left(\frac{{\left(p_{s}-p_{d}\right)}^{2}}{-2E_{p}^{2}}f\Delta t\right)}^{2}u^{2}\left(E_{p}\right)+{\left(\frac{{\left(p_{s}-p_{d}\right)}^{2}}{2E_{p}}\Delta t\right)}^{2}u^{2}\left(f\right)\\ \; & +{\left(\frac{{\left(p_{s}-p_{d}\right)}^{2}}{2E_{p}}f\right)}^{2}u^{2}\left(\Delta t\right){\}}^{\;1/2}. \end{array}$$

#### 4.2.3. AAA Growth

The growth $\Delta D=D_{i+1}-D_{i}$ measured between two successive examinations leads to the combined uncertainty

(9) $$\begin{array}{cc} u(\Delta D) & =\sqrt{u^{2}\left(D_{i+1}\right)+u^{2}\left(D_{i}\right)}=\sqrt{2}\cdot u\left(D\right). \end{array}$$

Normal values for blood pressure according to ESH guidelines are used as reference, a heart rate of 60 bpm, an AAA diameter of 30 mm, a 4.72 mm growth, a two-hour measuring interval, and the pressure–strain modulus determined at rest from the study by Länne [15,22,24]. With the given uncertainties, the combined uncertainties for dynamic load and the specific elastic deformation energy per measurement interval can be obtained (Table 1).

The uncertainties of the measured variables are given as standard deviation. Thus, the combined uncertainties for the load variable, the volume-specific deformation energy, and for aneurysm expansion are also standard deviations. The 95% confidence interval of the measurement uncertainty and the combined uncertainty is determined as 1.96·σ.

For the load variable, this implies that its absolute difference to the reference value must be at least $2.2\times 10^{7}$ and its relative difference to the reference value at least 57.2% in order to be outside the measurement uncertainty with a probability of 95%. The absolute difference of the volume-specific deformation energy to the reference value must be at least $1.67\times 10^{6}$ and the relative difference must be at least 122%. The measured growth of the aneurysm between two examinations must be at least 2.16 mm or 45.76% in relation to the reference value.

Varying systematically the systolic blood pressure and the heart rate while keeping the other input quantities constant, we obtain a contour plot for load and specific energy where we shade the area that lies outside the measurement uncertainty with a 95% level of confidence. It becomes clear that a systolic blood pressure greater than 140 mmHg shows a measurable effect in both load and specific energy. Furthermore, the load function is more sensitive to changes in heart rate than the specific energy function. This is because pulse pressure enters the energy function quadratically and therefore has a higher relative weight than the heart rate.

The quantification of the measurement uncertainty is important to determine whether the combined variables can be used in practice. That is, whether the correlations between load or energy, and the growth of the aneurysm can be detected at all within the given measurement uncertainty.

For the purposes of the study, this means that a measured growth of less than 2.16 mm in two consecutive examinations has no significance due to the measurement uncertainty (1.67×1.1) mm. The same applies to the measurement of the load and the energy entered between two examinations. If the increase in load or energy over the previous period is within the measurement uncertainty, it is not clearly due to changes in blood pressure, heart rate, or aneurysmal stiffness—it could be due to the measurement uncertainty.

To assess the implications, we can relate the combined measurement uncertainty of growth to typical expansion rates for aneurysms. Limet determined linear expansion rates of 5.3 mm/year for AAAs with a diameter smaller than 40 mm and of 6.9 mm/year for AAAs between 40 and 49 mm in size [25]. Vega determined mean growth rates of 2.07 mm/year for AAAs 30–39 mm in diameter and of 4.72 mm/year for AAAs 40–49 mm in diameter [15]. While the data in [25] were slightly skewed to the right, the data in [15] were normally distributed. Lederle determined similar growth rates [26]. It becomes clear that the expansion rate increases with aneurysm size [27]. Thus, examination intervals should be chosen with respect to aneurysm size, corresponding mean expansion rate, and the uncertainty of ultrasound measurement. If we consider a minimum growth measurement of 2.16 mm to lie outside measurement uncertainty 95% of the time as stated above, and assuming normally distributed expansion rates in study groups 1 and 2 (30–39 mm and 40–49 mm), appropriate examination intervals for both groups can be determined dividing *minimum expansion* by *expansion rate*.

Using the data from [15], we can relate examination intervals with the share of measured growth rates that lie outside measurement uncertainty. Choosing a smaller time interval between examinations would lead to an increasing number of AAAs with little progress where measured expansion lies within measurement uncertainty, while at the same time fast growing could be monitored more frequently. A threshold of 50%, i.e., half of the measurements fall outside measurement uncertainty, leads to an examination interval of 12 months for AAAs between 30 and 39 mm, and an interval of 6 months for AAAs between 40 and 49 mm. These intervals seem reasonable compared to regular surveillance intervals as stated in [27].

## 5. Discussion

How do the concepts of load input and specific energy input differ? The volume-specific, elastic deformation energy contains both the acting loads as input variables and the reaction of the vessel wall to the applied load. As a result, the pressure–volume modulus enters the energy function as stiffness parameter and the pulse pressure is weighted quadratically. Thus, the combined uncertainty of the specific energy is larger than that of the load function and at the same time it is more sensitive to changes in blood pressure. The maximum diameter and the growth rate correlate with the likelihood of rupture and are the most commonly used criteria in clinical practice. However, especially for aneurysms below the cut-off diameter of 50 mm and a growth rate of less than 10 mm/year, there is a need for additional criteria to assess their rupture risk.

Elevated blood pressure is already listed as a risk factor in the presence of an aneurysm, but little is known about the influence of heart rate on AAA evolvement. By introducing a load function and an energy function, we aim to quantify the influence of both blood pressure and heart rate and specifically take into account the dynamic nature of aneurysmal loading. Assuming a relationship between load input, specific energy input, and AAA evolvement, both functions could be used as biomechanical markers and complement established clinical criteria for rupture risk assessment. Furthermore, extensive blood pressure data allow us to analyze more precisely its influence on AAA progression. The significance of blood pressure peaks compared to mean arterial pressure could be studied, as well as the comparison of systolic and diastolic blood pressure.

It must be critically discussed in this context that the aforementioned aktiaa wristband only allows measurements of blood pressure at rest at a few defined times per day and that continuous blood pressure measurement is not yet possible. Nevertheless, the procedure proposed here is a step towards a much more comprehensive picture of vascular stress over a longer period of time.

In a first step, a prospective study could investigate whether the load input and the energy input correlate with AAA expansion rate as it is a measure known to be related to rupture risk and it can be easily determined in a routine examination. Additional biological markers associated with AAA progression and risk of rupture could be determined at every examination and tested for correlation with load and specific energy input. The most promising biomarker at present seems to be D-dimer concentration, a fibrin degradation product which correlates positively with AAA progression and is easy to determine as a routine parameter [28]. Care should be taken in the design of the study group and the collection of relevant control data that could influence the course of blood pressure, heart rate, and D-dimer concentration. Such a study will probably require a three-digit number of subjects and should therefore be designed as a multicenter trial. Aiming for a statistical power of β=0.8, a significance level of α=0.05, and assuming a correlation r=0.2 between growth rate ΔD and the biomarker dynamic loading *K*, a sample size of n=194 would be required.

## 6. Conclusions

Advancing technology in the optical measurement of heart rate and blood pressure via PPG offers the possibility of continuous tracking by handheld medical devices, smart watches, and fitness trackers. As mentioned above, other studies have already pointed out the influence of elevated blood pressure on aneurysm development. However, there are no approaches yet that take advantage of wearable blood pressure monitors to develop well-defined biomarkers based on physical vascular stress. Furthermore, previous approaches have never investigated the influence of heart rate on growth, although this correlates with increased cyclic stress on the vessel wall. Thus, we have the opportunity to study more precisely than ever before how these vital signs influence patient-specific AAA progression and rupture risk.

## Figures and Tables

**Table 1:** Reference values and uncertainties of the measurands and derived quantities. Pressure (mmHg), heart rate (bpm), diameter (mm), yearly growth (mm), stiffness (N/m^2^), volume-specific energy (J/m^3^), and relative uncertainty (%).

Measurand		Reference	Uncertainty	Rel. Uncertainty
	X	E(X)	u(X)	u(X)/E(X)
Blood pressure	$p_{s}$	120	7.75	6.64
	$p_{d}$	80	6.86	8.58
Heart rate	*f*	60	6.17	10.28
Diameter	*D*	30	0.78	2.6
Stiffness	$E_{p}$	$0.75\times 10^{5}$	$0.26\times 10^{5}$	34.67
Load	*K*	$3.84\times 10^{7}$	$1.12\times 10^{7}$	29.05
Energy	*W*	$1.37\times 10^{6}$	$0.85\times 10^{6}$	62.28
Growth	ΔD	4.7	1.1	23.3

## Data Availability

The data used to create the plots shown in this paper is referenced in the respective section.
